# Topological magnetoelectric response in ferromagnetic axion insulators

**DOI:** 10.1093/nsr/nwac138

**Published:** 2022-07-22

**Authors:** Yuhao Wan, Jiayu Li, Qihang Liu

**Affiliations:** Department of Physics and Shenzhen Institute for Quantum Science and Engineering (SIQSE), Southern University of Science and Technology, Shenzhen 518055, China; Department of Physics and Shenzhen Institute for Quantum Science and Engineering (SIQSE), Southern University of Science and Technology, Shenzhen 518055, China; Department of Physics and Shenzhen Institute for Quantum Science and Engineering (SIQSE), Southern University of Science and Technology, Shenzhen 518055, China; Shenzhen Key Laboratory of Advanced Quantum Functional Materials and Devices, Southern University of Science and Technology, Shenzhen 518055, China; Guangdong Provincial Key Laboratory for Computational Science and Material Design, Southern University of Science and Technology, Shenzhen 518055, China

**Keywords:** topological magnetoelectric effect, axion insulator, magnetic topological materials

## Abstract

The topological magnetoelectric effect (TME) is a hallmark response of the topological field theory, which provides a paradigm shift in the study of emergent topological phenomena. However, its direct observation is yet to be realized due to the demanding magnetic configuration required to gap all surface states. Here, we theoretically propose that axion insulators with a simple ferromagnetic configuration, such as the MnBi_2_Te_4_/(Bi_2_Te_3_)_n_ family, provide an ideal playground to realize the TME. In the designed triangular prism geometry, all the surface states are magnetically gapped. Under a vertical electric field, the surface Hall currents give rise to a nearly half-quantized orbital moment, accompanied by a gapless chiral hinge mode circulating in parallel. Thus, the orbital magnetization from the two topological origins can be easily distinguished by reversing the electric field. Our work paves the way for direct observation of the TME in realistic axion-insulator materials.

## Teaser

With appropriate geometry design, a ferromagnetic axion insulator is predicted to realize topological magnetoelectric response, which is urgently pursued as a hallmark phenomenon of the topological field theory.

## INTRODUCTION

The topological magnetoelectric effect (TME), e.g. the topological response of magnetization to an electric field in the same direction, is a hallmark phenomenon of the topological field theory [1,2]. Topological materials that possess a three-dimensional (3D) bulk axion field $\theta \ = \ \pi \ ( {{\mathrm{mod}}\ 2\pi } )$ accompanied by surface energy gaps are designated as axion insulators (AXIs) [1,3–7], which are expected to exhibit the TME. However, observing the TME is highly challenging owing to the requirement of introducing magnetic gaps on all the surfaces of a system with bulk $\theta \ = \ \pi $ [8–10]. The recent advent of intrinsic magnetic topological insulators (TIs), especially the MnBi_2_Te_4_/(Bi_2_Te_3_)_n_ family [11–25], has provided an ideal platform for the AXI phase. To date, the verification of the axion state has mostly focused on the measurement of the combined effect of two surfaces, say, the top and the bottom, such as the quantized Faraday/Kerr rotations [26–28] and the zero Hall plateau (ZHP) [9,29–32]. While the ZHP is conventionally regarded as a hallmark of AXIs, it is not exclusive to these, and ZHP signals may also appear in topological thin films gapped by quantum confinement and other non-axion cases [25,33–37]. Recent progress has focused on signals related to the surface anomalous Hall effect at a single gapped surface [37–43], which directly reveals the bulk-boundary correspondence of AXIs. Nevertheless, the TME, as the central assertion of the topological field theory, is yet to be observed.

**Figure 1.**
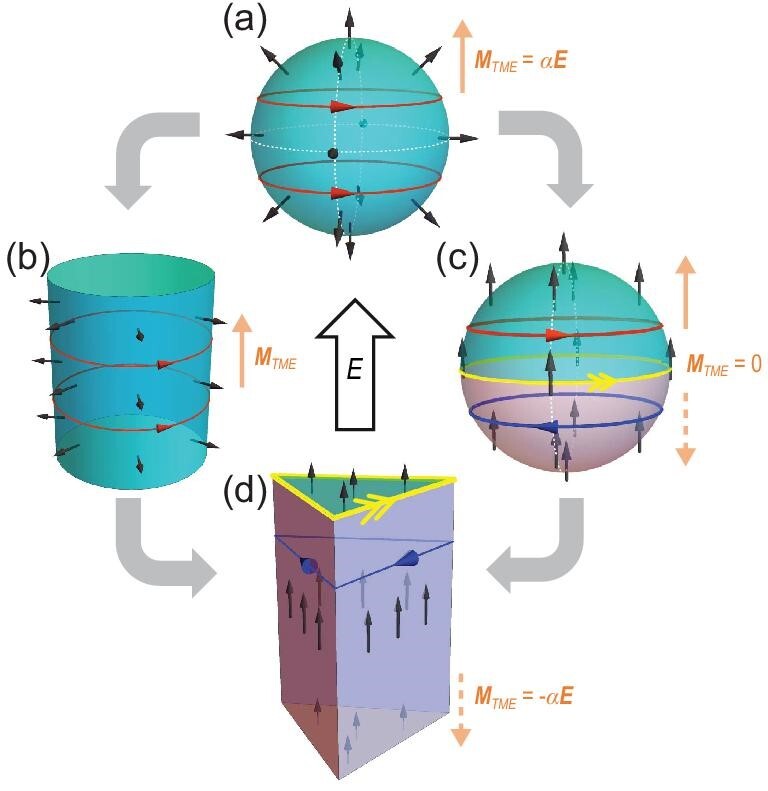
(a) Sketch of a 3D spherical TI with hedgehog-like surface magnetization (black arrows). By applying an electric field ${{\bf E}}$, the surface Hall effect gives rise to a circulating current (red lines) and thus generates a bulk magnetization ${{{\bf M}}}_{TME}\parallel {{\bf E}}$, with $\alpha = \frac{{{e}^2}}{{2hc}}$ as the fine structure constant. (b) The cylindrical TI with radial magnetic orbital moment; the same bulk magnetization can be induced by ${{\bf E}}$. (c) The spherical 3D TI with FM magnetization, where two hemispheres form two domains with a gapless chiral mode (yellow line). ${{\bf E}}$ cannot generate a net magnetization as the contributions from two domains compensate each other. (d) The triangular prism AXI, such as FM MnBi_2_Te_4_, where the side surfaces are gapped due to the hexagonal warping effect. A net ${{\bf E}}$-induced bulk magnetization is predicted.

The difficulty in realizing the TME mainly lies in the requirements of geometric and magnetic configurations, which are quite challenging for realistic materials. Historically, it was proposed that the TME could be realized in a spherical and strong TI with a hedgehog-like magnetization on its surface, or a cylindrical TI with a surface radial magnetization, as shown in Fig. 1(a) and (b), respectively [1,4,7]. Unfortunately, neither of these over-idealized configurations are accessible for experiments. It is conventionally believed that the TME can only be realized in a configuration without a net magnetic moment, e.g. antiferromagnetic (AFM) systems [4–6]. The reason is that a ferromagnetic (FM) AXI is typically a higher-order topological insulator (HOTI) simultaneously, accompanied by 1D hinge modes or magnetization-induced quantum Hall effects [see Fig. 1(c)] [1,44–47]. However, for AFM AXIs such as MnBi_2_Te_4_, the side surface state is gapless, protected by the combined symmetry of time reversal $\mathcal{T}$ and fractional translation ${\tau }_{1/2}$ [14,15], which also obscures the observation of the TME.

Here, we theoretically demonstrate that by designing particular configurations, an FM AXI, such as MnBi_2_Te_4_/(Bi_2_Te_3_)_n_, can serve as an ideal platform for realizing a topological magnetoelectric response. The simple magnetic configuration, which could be achieved directly in MnBi_8_Te_13_ with an FM ground state [21,40] and in MnBi_2_Te_4_, MnBi_4_Te_7_ and MnBi_6_Te_10_ (AFM ground state) [13,16,20,22] under a moderate vertical magnetic field, ensures the accessibility of our proposal. Remarkably, the side surface states of such FM AXIs manifest magnetic gaps owing to the hexagonal warping effect [19,48]. In the designed geometry of the triangular prism [see Fig. 1(d)], a chiral hinge mode from the HOTI phase is pinned to circulate around the top surface rather than the entire bulk. When an electric field is applied along the prism, the side surface Hall current also circulates parallel to the top surface, thus avoiding interference with the hinge mode. We calculate the bulk magnetization as a response to the external electric field and obtain the nearly half-quantized response coefficient. In a realistic finite-layer system, the TME-induced magnetization can be directly extracted by reversing the electric field, as the signals from the TME and hinge mode are odd and even under field reversal, respectively. Moreover, we characterize the thickness-driven crossover of the FM AXI from 3D HOTI to 2D Chern insulator by visualizing the distribution of the chiral mode. Our findings provide an accessible material-based proposal for achieving the long-sought TME to validate the topological field theory.

## RESULTS AND DISCUSSION

### Configuration for realizing topological magnetoelectric effect

According to the topological field theory, the gradient of the static axion field $\nabla \theta $ at the surfaces of TIs leads to the TME through the surface half-quantized Hall current [1,3]. Imagining a 3D time-reversal invariant TI with surface states gapped homogeneously by spatially oriented surface magnetic moments [see Fig. 1(a) and (b)], the surface Hall current density induced by the external electric field ${{\bf E}}$ is


(1) \begin{eqnarray*}
{{{\bf j}}}_H = \frac{\alpha }{\pi }{{\bf E}} \times \nabla \theta ,\
\end{eqnarray*}

with $\alpha = {e}^2/2hc$ as the fine structure constant. Such a circulating current contributes to the TME, i.e. an emergent magnetization parallel to ${{\bf E}}$ with a quantized coefficient ${{{\bf M}}}_{TME} = \alpha {{\bf E}}$. Re-examining the necessity of such impractical spatially oriented magnetization, we find that under FM moments, the shell of the spherical TI is divided into two domains with opposite magnetic surface gaps forming a gapless chiral mode at the domain wall. In most cases, dissipation occurs when the circulating currents encounter the gapless chiral mode, violating the adiabatic condition of the TME, i.e. the side surface states should be gapped to ensure the circulation of the Hall currents [1,9]. However, if the chiral mode is pinned to circulate around a 2D plane, interference between the TME and the chiral mode would be avoided if the electric field is applied perpendicular to the plane. Although in Fig. 1(c) the surface Hall currents from the two hemispheres compensate each other, leading to a zero net magnetoelectric response, FM systems could potentially reveal the TME by designing inequivalent domains.

Exemplified by MnBi_2_Te_4_/(Bi_2_Te_3_)_n_, we propose a material-based structure to realize the TME in an FM AXI with triangular prism geometry, as shown in Fig. 1(d). Previous studies have shown that the MnBi_2_Te_4_ family of out-of-plane FM ordering are both AXIs protected by inversion symmetry and HOTIs [19,21,41]. In a hexagonal prism sample, the six side surfaces are gapped by staggered mass terms with respect to the 3-fold rotation and inversion symmetries [19,46]. The gapless chiral modes at the domain walls of the side surfaces thus obscure the measurements of the TME. However, once the AXI adopts a triangular prism configuration, all three side surfaces have the same sign of magnetic surface gaps, which is coincident with that of, say, the bottom surface. Therefore, the gapless chiral mode is localized only at the top surface, rather than circulating around the entire bulk. Once the vertical electric field is applied, the responding Hall current travels parallel to the hinge mode without any interference, leading to a measurable magnetization (anti)parallel to the electric field. Recently, the HOTI bismuth with similar helical hinge modes [49] was successfully synthetized in a triangular geometry [50]. This implies that our proposal for realizing the TME in the FM MnBi_2_Te_4_ family is experimentally accessible.

### Model for ferromagnetic axion insulator MnBi_2_Te_4_

To calculate the TME of an FM AXI, we start from the effective model Hamiltonian of FM MnBi_2_Te_4_ written in a triangular lattice [19,42]


(2) \begin{eqnarray*}
\mathcal{H} = {d}_0{I}_4 + \mathop \sum \limits_{i{\mathrm{ }} = {\mathrm{ }}1, \cdots ,5} {d}_i{{\mathrm{\Gamma }}}_i + {\mathrm{\Delta }}{{\bf m}} \cdot {{\bf s}} \otimes {\sigma }_0,{\mathrm{\ }}
\end{eqnarray*}

where ${d}_0 = \tilde{C} - 2{C}_1\cos {k}_z - ( {4{C}_2/3} )( \cos {k}_1 + \cos {k}_2 + \cos {k}_3 )$, ${d}_1 = ( {v/3} )( 2\sin {k}_1 + \sin {k}_2 + \sin {k}_3 )$, ${d}_2 = ( {v/\sqrt 3 } )( {\sin {k}_2 - \sin {k}_3} )$, ${d}_3 = {v}_z\sin {k}_z$, ${d}_4 = 8w(\!{ -\! \sin {k}_1 + \sin {k}_2 + \sin {k}_3} )$ and ${d}_5 = \tilde{M} - 2{M}_1\cos {k}_z - ( {4/3} ){M}_2( \cos {k}_1 + \cos {k}_2 + \cos {k}_3 )$ with $\tilde{R} = {R}_0 + 2{R}_1 + 4{R}_2$ ($R{\mathrm{\ }} = {\mathrm{\ }}C,M$), ${k}_1 = {k}_x$, ${k}_2 = ( {{k}_x + \sqrt 3 {k}_y} )/2$ and ${k}_3 = {k}_1 - {k}_2$. Here ${I}_4$ is the identity matrix, ${{\mathrm{\Gamma }}}_i = {s}_i \otimes {\sigma }_1{\mathrm{\ }}$ for $i{\mathrm{\ }} = {\mathrm{\ }}1,2,3$, ${\mathrm{\ }}{{\mathrm{\Gamma }}}_4 = {s}_0 \otimes {\sigma }_2$ and${\mathrm{\ }}{{\mathrm{\Gamma }}}_5 = {s}_0 \otimes {\sigma }_3$, where ${s}_i$ and ${\sigma }_i$ are the Pauli matrices for spin and orbital, respectively. $v{\mathrm{\ }}( {{v}_z} )$ represents the velocity along the in-plane (out-of-plane) direction, *w* is the hexagonal warping parameter [48,51], ${\mathrm{\Delta }}$ describes the exchange coupling between the electron states and magnetic moments and ${{\bf m}} = ( {0,0,1} )$ stands for the out-of-plane FM order. The model parameters are presented in Supplementary Data Section 1.

**Figure 2.**
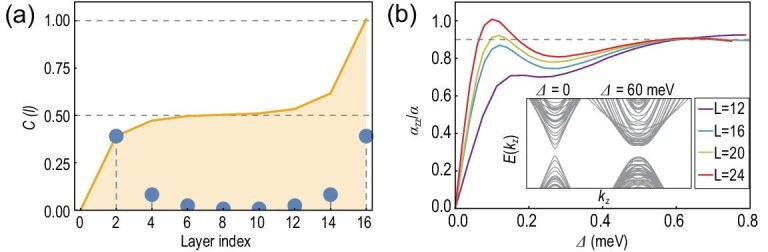
(a) Layer-resolved Chern number of a 16-layer triangular prism FM MnBi_2_Te_4_. Blue points represent the local Chern number $\mathbb{C}( i )$ for each layer, and the orange line shows the integrated Chern number $C( l ) = \mathop \sum \nolimits_{i = 1}^l \mathbb{C}( i )$ over the layers. (b) Magnetoelectric coefficient as a function of the exchange coupling ${\mathrm{\Delta }}$ in triangular prism FM MnBi_2_Te_4_, with different side length *L* and translation symmetry along the *z*-direction. Inset: band structures of the $L = 14$ configuration with different ${\mathrm{\Delta }}$.

The model Hamiltonian of FM MnBi_2_Te_4_ respects 3-fold rotation around the *z*-axis ${C}_{3z} = {e}^{i\pi {s}_3/3} \otimes {\sigma }_0$ and inversion $\mathcal{P} = {s}_0 \otimes {\sigma }_3 = {{\mathrm{\Gamma }}}_5$. Consequently, the bulk topology of this inversion-preserved system can be described by the symmetry indicator of $\mathcal{P}$, i.e. ${\mathbb{Z}}_4 \times {\mathbb{Z}}_2 \times {\mathbb{Z}}_2 \times {\mathbb{Z}}_2$ [52–55]. We thus verify that the FM MnBi_2_Te_4_ is an FM AXI with a symmetry indicator (2;000) (Supplementary Data Section 1). Such an AXI phase can also be confirmed by calculating the real-space resolved Chern marker [56–58]. In Fig. 2(a) we show that in the 16-slab model, the top and bottom four layers contribute a nearly half-quantized Chern number ${C}_{t( b )} = \pm 1/2$, indicating the bulk topology $\theta \ = \ \pi $. In addition, owing to the hexagonal warping term ${d}_4$, the side surface states open a magnetic gap (Supplementary Data Section 1). Previous first-principles calculations show that in FM MnBi_2_Te_4_ such a high-order magnetic gap is ∼6 meV [41], which is typically larger than the finite-size gap of the few-layer slabs. Since the top and bottom surface states are gapped by the exchange coupling, all the surfaces of the configuration are gapped. Due to the ${C}_{3z}$ symmetry, the gap signs of the side surfaces are the same and coincide with either that of the top surface or the bottom surface (Supplementary Data Section 1). In this work, $( {100} ),( {0\bar{1}0} ),( {\bar{1}10} )$ surfaces are chosen as the side surfaces, of which the bottom surface shares the same gap sign.

### Topological magnetoelectric response

To evaluate the TME, we next calculate the orbital magnetization induced by the vertical electric field through linear response theory. By constructing an equilateral triangle lattice in the $xy$-plane with a side length of *L* while maintaining the translation symmetry along *z*, we obtain the field-induced orbital magnetization ${M}_{TME,i} = {\alpha }_{ij}{E}_j$, in which the only relevant coefficient component ${\alpha }_{zz}$ is calculated via the Kubo formula [59]


(3) \begin{eqnarray*}
{\alpha }_{zz} &=& \frac{{ - i}}{V}\mathop {\lim }\limits_{{\mathrm{\Omega }} \to 0} \mathop \int \nolimits_{ - \infty }^{ + \infty } dt^{\prime}{e}^{i{\mathrm{\Omega }}\left( {t - t^{\prime}} \right)}\theta ( {t - t^{\prime}} )\\
&&\times \langle[ {{{\hat{M}}}_z\left( t \right),{{\hat{J}}}_z( {t^{\prime}} )} ]\rangle,
\end{eqnarray*}

where *V* is the system volume, $\theta ( x )$ is the step function and ${\mathrm{\Omega }}$ is the frequency of the electric field. $\langle O\rangle = {\mathrm{Tr}}[ {{e}^{ - \beta H}O} ]/{\mathrm{Tr}}{e}^{ - \beta H}$ denotes the ensemble average, with $\beta $ the inverse temperature. The orbital magnetic moment is defined as ${{\bf \hat{M}}} = - ( {e/2c} ){{\bf \hat{r}}} \times {{\bf \hat{v}}}$ [60], with ${{\bf \hat{r}}}$ and ${{\bf \hat{v}}}$ the position and velocity operators, respectively; the current density is ${{\bf \hat{J}}} = e{{\bf \hat{v}}}$. In the zero-temperature limit with the Fermi level ${\mu }_F$ lying inside the band gap, Equation (3) can be simplified as follows [61]


(4) \begin{eqnarray*}
{\alpha }_{zz} &=& \frac{{2\hbar }}{V}\mathop \sum \limits_{{k}_z} \mathop \sum \limits_{\begin{array}{@{}*{1}{c}@{}}\\\scriptstyle{i \in occ.}\\ \scriptstyle{j \in unocc.} \end{array}}\\
&&\times \frac{{Im\left[ \langle{{k}_z,\!i{\mathrm{|}}{{\hat{M}}}_z{\mathrm{|}}{k}_z,\!\!j\rangle\langle {k}_z,\!\!j{\mathrm{|}} {\hat{{J}}}_z{\mathrm{|}}{k}_z,\!i}\rangle \right]}}{{{{\left( {{\varepsilon }_{i,{k}_z} - {\varepsilon }_{j,{k}_z}} \right)}}^2}},\\
\end{eqnarray*}

where $|{k}_z,\!i\rangle $ is the *i*-th eigenstate with momentum ${k}_z$ with respect to the eigenvalue ${\varepsilon }_{i,{k}_z}$. During the summation, *i* and *j* are constrained within the occupied and unoccupied bands, respectively.

The numerical results of the magnetoelectric response coefficient ${\alpha }_{zz}$ for different side lengths *L* are shown in Fig. 2(b). If the system size is large enough ($L \ge \ 12$), ${\alpha }_{zz}$ approaches a nearly quantized value $0.9\alpha $ with an exchange coupling ${\mathrm{\Delta \ }} = \ 62$ meV. This is the central result of our work. To verify our theoretical approach and the topological origin of ${\alpha }_{zz}$, we tune the parameter ${M}_0$ in ${d}_5$ to drive the MnBi_2_Te_4_ bulk to the $\theta \ = \ 0$ side, and find that the corresponding ${\alpha }_{zz}$ vanishes accordingly (Supplementary Data Section 2). According to the $\theta \ = \ \pi $ nature in a bulk AXI, we can expect a quantized ${\alpha }_{zz} = \alpha $ in a large-size AXI sample with all the surface gapped, while the symmetry of the bulk axion field (e.g. inversion) is preserved. This can be verified by calculating the TME of an ideal 3D AXI protected by both $\mathcal{P}$ and $\mathcal{T}$, with a cubic lattice and radial side surface magnetization (Supplementary Data Section 2). Even $\mathcal{T}$ symmetry is broken by the surface magnetization. The finite tetragonal structure preserves $\mathcal{P}$ symmetry and we find that ${\alpha }_{zz}$ approaches to $\alpha $ for increasing *L*. Therefore, in our FM AXI configuration, the deviation between the saturation value $0.9\alpha $ and the perfect quantization ($\alpha $) is attributed to the inversion breaking of the triangular prism.

The profile of ${\alpha }_{zz}$ experiences a steep increase followed by oscillation and eventually saturation as the magnetization ${\mathrm{\Delta }}$ increases from zero. To understand this, we plot the band spectra along ${k}_z$ for different ${\mathrm{\Delta }}$ values, as shown in the inset of Fig. 2(b). Without magnetization (${\mathrm{\Delta \ }} = \ 0$), the band gap, i.e. the side surface gap of the triangular prism, originates from the finite-size effect of the in-plane triangular geometry characterized by *L*, whereas for ${\mathrm{\Delta \ }} = \ 70\ {\mathrm{meV}}$ the gap is dominated by magnetization after a non-trivial phase transition. Here the non-trivial topology of this finite-size system is characterized by the topological magnetoelectric coefficient, i.e. non-zero Hall current induced by the external electric field. Although there is no gap closing as ${\mathrm{\Delta }}$ increases, the phase transition can be monitored by the change in the local Chern marker of the side surface (Supplementary Data Section 3), thus giving rise to the evolution of ${\alpha }_{zz}$. As *L* increases, ${\alpha }_{zz}$ grows more rapidly when turning on ${\mathrm{\Delta }}$. This is because the finite-size-induced hybridization gap reduces and thus becomes more easily overwhelmed by the magnetic gap.

**Figure 3.**
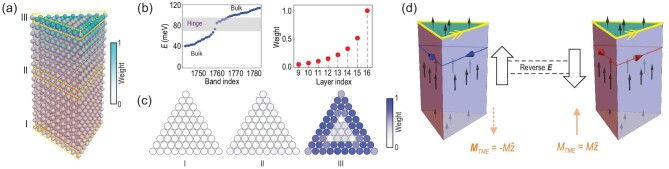
(a) Lattice model of the triangle prism FM MnBi_2_Te_4_ for side length $L = 10$ and height $H = 16$. (b) Left panel: energy levels of the lattice model, with the eigenenergies of the hinge modes highlighted by gray shades. Right panel: layer distribution of one of the hinge modes. (c) In-plane distribution of the hinge mode for different layers in (a). (d) Switching the direction of the electric field reverses the Hall current and the sign of ${{{\bf M}}}_{TME}$, while the itinerant circulation magnetization that originated from the chiral hinge mode remains intact.

In realistic samples, the translation symmetry along the *z* direction is broken and the prism is terminated by the top and bottom surfaces, which have a larger magnetic gap than those of the side surfaces. Consequently, the top surface carries a gapless chiral hinge mode [see Fig. 1(d)], while there is no hinge mode propagating along the *z* direction. We consider several prism models with different heights of *H* layers and directly diagonalize them, and find that the energy of the hinge state converges as $H > 10$. The distribution of the corresponding wavefunction decays exponentially from the hinge (Supplementary Data Section 4). Figure 3(a–c) shows the prism geometry, energy level and distribution of the hinge state of the FM AXI with $L = 10$ and $H = 16$. Such a hinge state does contribute an extra orbital magnetization ${M}_{IC} \simeq - e{\mu }_F/\hbar c$, which is also known as itinerant circulation magnetization [60,62].

Next, we discuss how to distinguish the TME-induced orbital moment from the total magnetization. Since there is no interference between the surface Hall current and the chiral hinge mode, the total bulk magnetization ${M}_{tot}$ is a superposition of the local moment ${M}_{ion} = {n}_i\langle{S}_{{\mathrm{Mn}}}\rangle $ from Mn ions (${n}_i$ and $\langle{S}_{{\mathrm{Mn}}}\rangle $ are the density and average spin of Mn ions, respectively), itinerant circulation magnetization ${M}_{IC} \simeq - e{\mu }_F/\hbar c$ from the chiral hinge state, and the TME-induced magnetization ${M}_{TME} = {\alpha }_{zz}{E}_z$ from the surface Hall current. Both ${M}_{ion}$ and ${M}_{IC}$ are independent on the vertical electric field (up to the first order, before it leads to a topological phase transition), whereas ${M}_{TME}$ is an odd function of ${{\bf E}}$. Therefore, the net TME signal can be easily extracted via two measurements with opposite electric fields ${M}_{TME} = [ {{M}_{tot}( {{\bf E}} ) - {M}_{tot}( { - {{\bf E}}} )} ]/2$, as illustrated in Fig. 3(d).

### Magnetic gap versus hybridization gap

**Figure 4.**
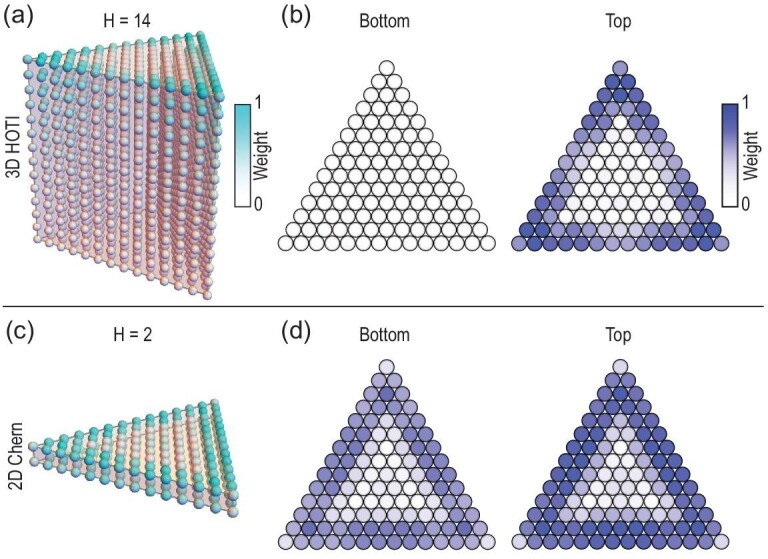
(a) Lattice of a 14-layer triangle prism FM MnBi_2_Te_4_ as a 3D HOTI. (b) The distributions of the gapless hinge modes projected on the bottom and top layers of (a), respectively, indicating a well-localized hinge state. (c) Lattice of a bilayer structure of the same material, manifesting a 2D Chern insulator phase. (d) Projection of the gapless edge state on the bottom and top layers.

For a thin slab (small *H*), the hinge mode encounters the bottom surface before it completely evanesces, leading to a crossover between 3D HOTI and 2D Chern insulator, as shown in Fig. 4. Both the HOTI and Chern insulator phases manifest half-quantized anomalous Hall conductivities at the top and bottom surfaces [4,41]. The crossover between such 3D and 2D topological matter is determined by the gap nature of the side surface, i.e. the competition between magnetization and finite-size hybridization. With a large enough *H*, the magnetic gap of the side surface ensures a chiral hinge mode ($\frac{1}{2} + \frac{1}{2}$) at the top surface but nothing ($\frac{1}{2} - \frac{1}{2}$) at the bottom surface [see Fig. 4(a) and (b)]. In comparison, as *H* reduces, for all side surfaces, the gap gradually becomes a hybridization gap, leading to the localization of the half-quantized anomalous Hall conductivity at the top and bottom surfaces, as shown in Fig. 4(c) and (d).

For comparison, we also calculate the TME of the AFM AXI exemplified by A-type AFM MnBi_2_Te_4_ with the Néel vector oriented along the *z*-direction, manifesting a gapless Dirac-like side surface state because of the protection of $\mathcal{T}{\tau }_{1/2}$ [14,15]. For a finite sample, the side surface gap is always a hybridization gap. Our calculations show that the TME coefficient is always zero as a function of the exchange coupling (Supplementary Data Section 5). The comparison between the vanishing TME in the AFM AXI and the non-zero TME in the FM AXI reveals the importance of the surface magnetic gap in generating the topological response. We note that the realization of the TME has been proposed in AFM magnetic heterostructures and MnBi_2_Te_4_ models under an in-plane electric field, which generates surface Hall currents circulating through the magnetic-gapped top and bottom surfaces and hybridization-gapped side surfaces [9,10,63]. However, it might be challenging to simultaneously maintain an appropriate surface gap size (requires a thin slab) and avoid interference between surface anomalous Hall currents from the top and bottom surfaces (requires a thick slab).

## CONCLUSION

To summarize, we demonstrate that the inversion-preserved FM AXIs MnBi_2_Te_4_/(Bi_2_Te_3_)_n_ serve as a realizable platform for achieving the topological magnetoelectric response. Designed in a triangular prism geometry, the side surfaces are gapped by a uniform FM exchange field instead of the elaborately oriented magnetic moments in previous studies. Using linear response theory, we obtain nearly half-quantized orbital magnetization induced by a vertical electric field. In a realistic finite-layer sample, the TME signal can be directly extracted from the itinerant circulation magnetization and local ion moments by reflecting the electric field. In parallel, an electric polarization induced by a time-dependent vertical magnetic field could also be expected in our configuration. Given the enormous number of observations verifying the successful topological band theory, our findings provide an accessible proposal for achieving the long-sought TME in realistic materials, which sheds light on more response properties predicted by the topological field theory.

## Supplementary Material

nwac138_Supplemental_File
