# Supplementary material for: Topological magnetoelectric response in ferromagnetic axion insulators
Source: Natl Sci Rev. 2022 Jul 22;11(2):nwac138. doi: 10.1093/nsr/nwac138 (PMC10804227; doi:10.1093/nsr/nwac138)
Supplement: nwac138_Supplemental_File [file nwac138_supplemental_file.docx]

**Supplementary data**

**Topological Magnetoelectric Response in Ferromagnetic Axion Insulators**

Yuhao Wan^1^, Jiayu Li^1^, and Qihang Liu^1,2,3,*^

*^1^Department of Physics and Shenzhen Institute for Quantum Science and Engineering (SIQSE), Southern University of Science and Technology, Shenzhen 518055, China*

*^2^Guangdong Provincial Key Laboratory for Computational Science and Material Design, Southern University of Science and Technology, Shenzhen 518055, China*

*^3^Shenzhen Key Laboratory of Advanced Quantum Functional Materials and Devices, Southern University of Science and Technology, Shenzhen 518055, China*

Y.W. and J.L. contributed to the work equally.

^*^Email: [liuqh@sustech.edu.cn](mailto:liuqh@sustech.edu.cn)

**Contents**

[**1. Model of ferromagnetic MnBi_2_Te_4_** 2](#_Toc84934244)

[**2. Topological magnetoelectric effect for different structures** 5](#_Toc84934245)

[**3. Transition between surface hybridization gap and magnetic gap** 7](#_Toc84934246)

[**4. Finite-layer model of ferromagnetic MnBi_2_Te_4_** 8](#_Toc84934247)

[**5. Magnetoelectric response in antiferromagnetic MnBi_2_Te_4_** 9](#_Toc84934248)

# **1. Model of ferromagnetic MnBi_2_Te_4_**

The effective model of ferromagnetic (FM) MnBi_2_Te_4_ is provided as Eq. (2) in the main text. The topology of this lattice model is described by the symmetry indicator of $\mathcal{P}$: $\mathbb{Z}_{4}\times\mathbb{Z}_{2}\times\mathbb{Z}_{2}\times\mathbb{Z}_{2}$, where the $\mathbb{Z}_{4}$ and $\mathbb{Z}_{2}$ indices are defined as

$$\mathbb{Z}_{4}=\sum_{\mathbf{k}\in I} \frac{n_{\mathbf{k}}^{+}-n_{\mathbf{k}}^{-}}{2} \mathrm{mod} 4, \mathbb{Z}_{2}^{\left( j \right)}=\sum_{\mathbf{k}\in I_{j}} n_{\mathbf{k}}^{\boldsymbol{-}} \mathrm{mod} 2, \left( S1 \right)$$

where $I=\left\{ \sum_{i=1}^{3} \frac{n_{i}}{2}\mathbf{b}_{i}|n_{i}=0,1 \right\}$ is the set of eight inversion-invariant momenta with $\mathbf{b}_{i}$ the reciprocal lattice vectors, $I_{j}=\left\{ \sum_{i=1}^{3} \frac{n_{i}}{2}\mathbf{b}_{i}|n_{j}=1,n_{i\left( \neq j \right)}=0,1 \right\}$ are subsets of $I$, and $n_{\mathbf{k}}^{+/-}$ are the number of occupied states with even/odd parity at momentum $\mathbf{k}$. Distinct phases can be achieved by tuning the ratio between $\Delta$, $M_{0}$, and $M_{1,2}$. Without loss of generality, we choose $M_{1}=M_{2}=0.1$eV and obtain different band topology via tuning $\Delta$ and $M_{0}$. The phase diagram of the FM MnBi_2_Te_4_ model is shown in Fig. S1, with the corresponding symmetry indicators $\left( \mathbb{Z}_{4}:\mathbb{Z}_{2}\mathbb{Z}_{2}\mathbb{Z}_{2} \right)$ indicated.

According to the phase diagram, we set $M_{0}=-0.2 eV,M_{1}=0.1 eV,M_{2}=0.1 eV,C_{0}=0,C_{1}=30 meV,C_{2}=40 meV,v_{z}=0.1 eV, v=50 meV, and w=37.5 \mathrm{meV}$ throughout the calculation for the inversion-protected FM axion insulator (AXI) phase [1].

The side surfaces of the FM AXI are gapped by the joint effect of the hexagonal warping and out-of-plane magnetization ($\Delta s_{z}$). To verify this, we start from the Hamiltonian of the (010) surface state, up to the cube of $\mathbf{k}$ [1]

$$\mathcal{H}_{\left( 010 \right)}\left( k_{x},k_{z} \right)=\tilde{v}k_{x}\sigma_{z}+\tilde{v}_{z}k_{z}\sigma_{x}-\frac{\Delta C_{1}}{M_{2}}\sigma_{z}+\tilde{w}k_{x}^{3}\sigma_{y}. \left( S2 \right)$$

with $\tilde{r}\propto r$ ($r=v,v_{z},w$) the dressed parameters. After a translation transformation: $k_{x}\to k_{x}+\Delta C_{1}/\tilde{v}M_{2}$, we obtain a side surface gap of $\varepsilon_{g}=\tilde{w}C_{1}^{3}\Delta^{3}/\tilde{v}^{3}M_{2}^{3}$, in which the gap size is about 6 meV observed in first principle calculation [2].


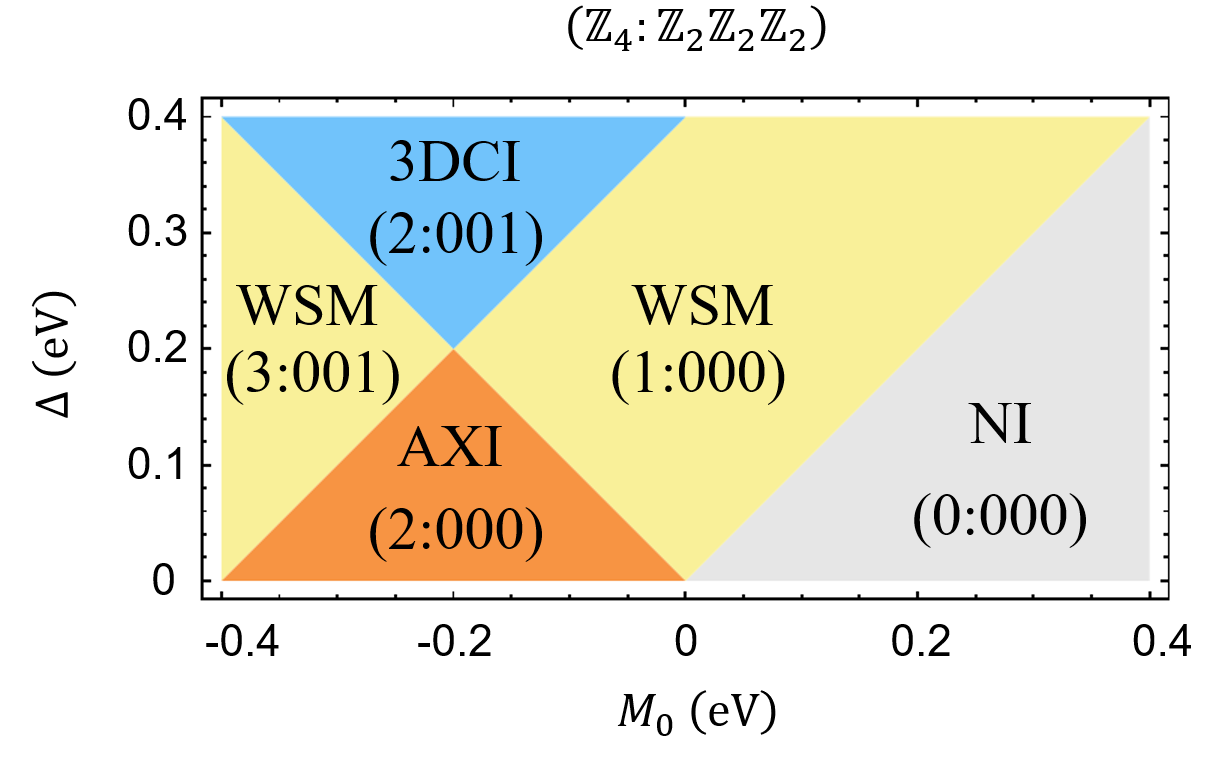


**Fig. S1.** Phase diagram of FM MnBi_2_Te_4_, including normal insulator (NI), Weyl semimetal (WSM), axion insulator (AXI), and 3D Chern insulator (3DCI) phases.

In order to generate the topological magnetoelectric effect (TME) in the FM MnBi_2_Te_4_, the configuration (geometry) of the material should be elaborated. Even the FM MnBi_2_Te_4_ are known as inversion-protected AXI, the material of the hexagonal prism geometry cannot generate the TME with the chiral hinge mode around the edges of the sample [see Fig. S2(a)]. As the chiral mode results from the opposite surface magnetic gap of the adjacent surfaces, the directions of the Hall current of two surfaces are antiparallel when the electric field is applied along the prism. Hence, there is no circulating Hall current to generate the TME in this geometry. This vanishing TME can also be considered as the interference between the chiral mode and the surface Hall current, since the gapless chiral mode violates the full surface gap condition, *i.e.*, “adiabatic condition”.


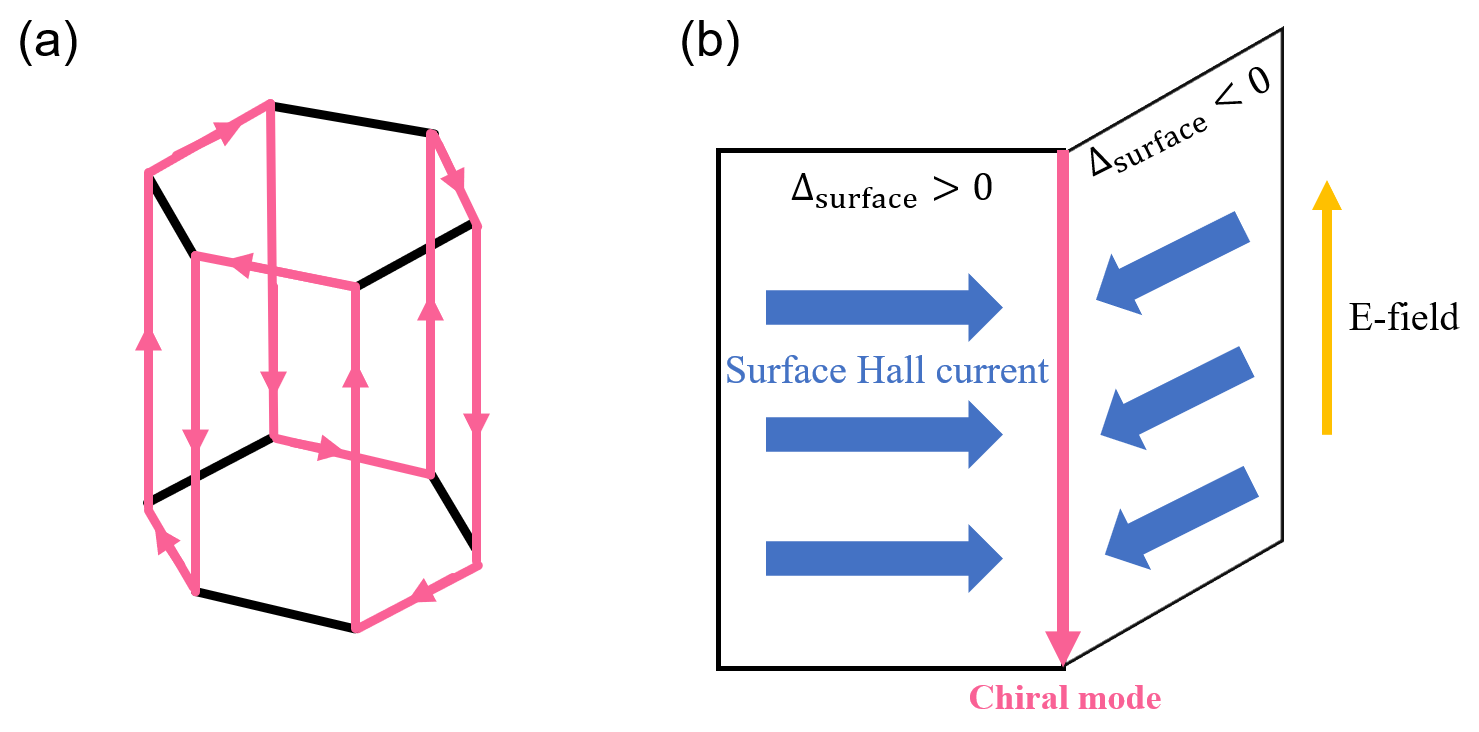


**Fig. S2.** (a) Chiral hinge mode of the ferromagnetic MnBi_2_Te_4_ of hexagonal prism geometry, cited from Ref. [1]. (b) Interference between the surface Hall current and the chiral hinge mode.

To avoid the interference between the chiral mode and the surface Hall current, the configuration can be designed in a triangular prism as sketched in Fig. 1(d) of the main text. As the hexagonal prism of AXI with FM order is protected by $C_{3z}$ and $M_{x}\mathcal{T}$ symmetries, we find that the surface magnetic gap of the $\left( 1\bar{1}0 \right)$ and $\left( \bar{1}00 \right)$ surfaces are same as the gap in $\left( 010 \right)$, while the other three side surfaces $\left( 100 \right),\left( 0\bar{1}0 \right),\left( \bar{1}10 \right)$ share the same magnetic gap, as shown in Fig. S3(a).


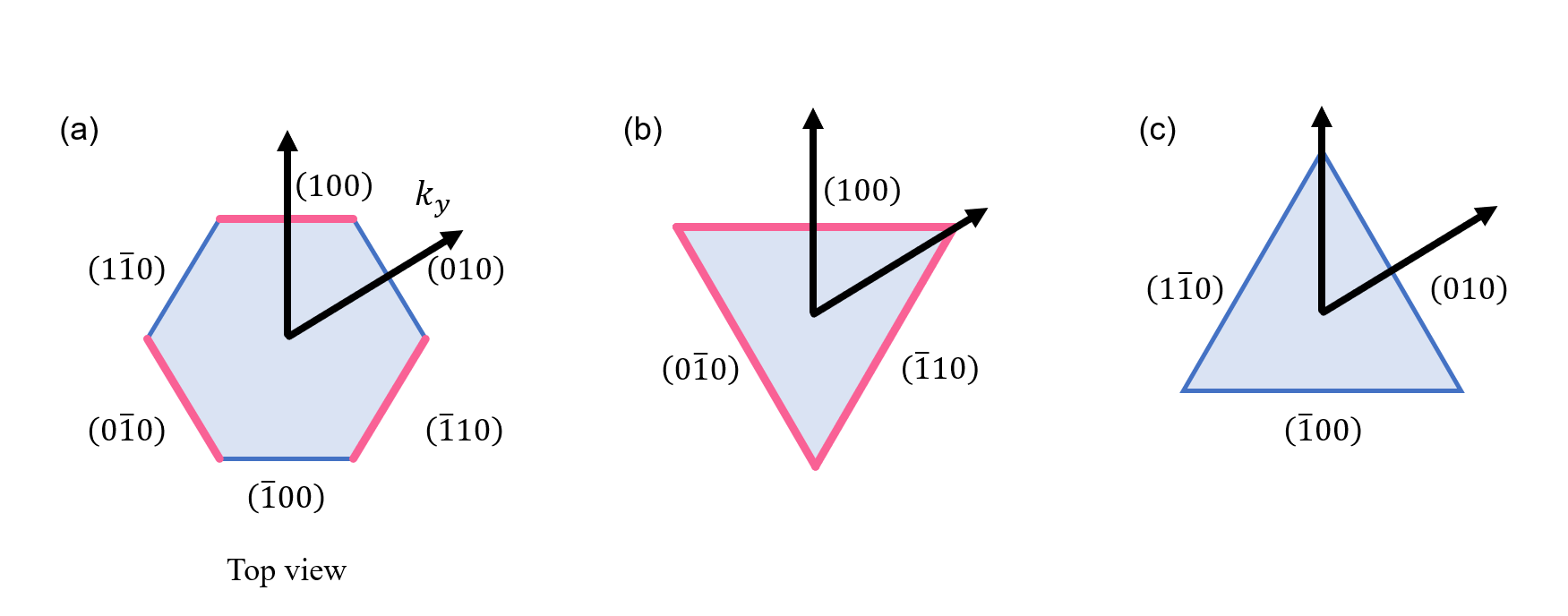


**Fig. S3.** (a) Top view of chiral mode and six side surfaces of the hexagonal prism ferromagnetic MnBi_2_Te_4_ with alternant surface magnetic gaps. (b) Triagonal prism with chiral mode around the hinge between the top surface and $\left( 100 \right),\left( 0\bar{1}0 \right),\left( \bar{1}10 \right)$ surfaces. (c) There is no chiral mode seen through the top view, as the chiral mode present at the hinge between the bottom surface and $\left( 010 \right),\left( 1\bar{1}0 \right),\left( \bar{1}00 \right)$ surfaces.

Because the material is FM with magnetic order pointing $+z$ direction, the surface Dirac cones of the top/bottom surfaces are gapped by the term $\propto\pm\Delta\sigma_{z}$, since the projection of the magnetic moment on the surface normal vector $\hat{\mathbf{n}}$ are opposite (${\hat{\mathbf{n}}}_{\mathrm{top}}\boldsymbol{=}\hat{z}=-{\hat{\mathbf{n}}}_{\mathrm{bottom}}$). Then the top surface and the $\left( 010 \right),\left( 1\bar{1}0 \right),\left( \bar{1}00 \right)$ side surfaces have the same sign of positive magnetic gap, while the bottom surface and the other three side surfaces take the negative gap. Now if we reduce the area of $\left( 010 \right),\left( 1\bar{1}0 \right),\left( \bar{1}00 \right)$ terminations but preserve the $C_{3z}$ symmetry, the remaining three side surfaces take the opposite magnetic gap with respect to the top surface. Then the chiral mode emerges at the boundary of magnetic gap domains, as shown in Fig. S3(b). On the contrary, the chiral mode will emerge at the hinge between the bottom surface and side surfaces once the $\left( 100 \right),\left( 0\bar{1}0 \right),\left( \bar{1}10 \right)$ surfaces are reduced [Fig. S3(c)]. Therefore, the location of the chiral mode depends on the crystal orientation of the side surfaces.

# **2. Topological magnetoelectric effect for different structures**

We first plot the magnetoelectric response $\alpha_{zz}$ for different phases of FM MnBi_2_Te_4_. As depicted in Fig. S1, FM MnBi_2_Te_4_ turns into a normal insulator (NI) phase when $M_{0}=0.1 \mathrm{eV}$, for which the magnetoelectric response vanishes. In Fig. S4 we display the numerical result of magnetoelectric response coefficient of NI phase. In contrast to the nearly half-quantized coefficient in the AXI phase [see Fig. 2(b) in main text], the magnetoelectric response is absent in the NI phase.


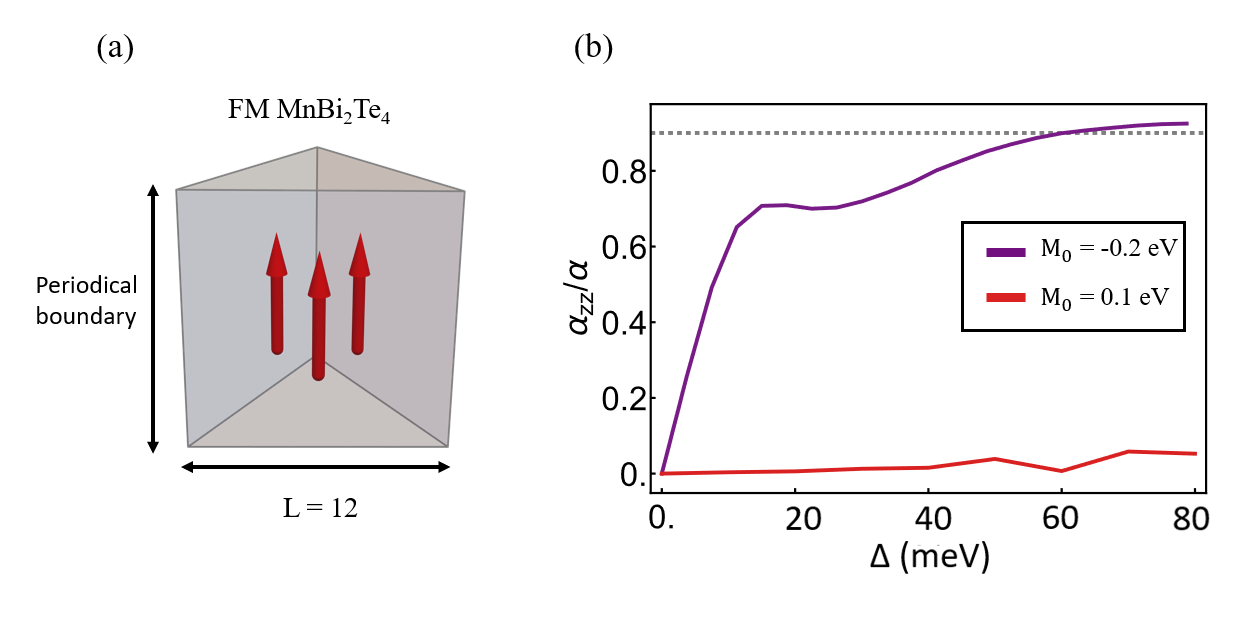


**Fig. S4.** (a) Sketch of triangular prism FM MnBi_2_Te_4_ with side length $L=12$. (b) Magnetoelectric coefficient as a function of the exchange coupling $\Delta$ in triangular prism FM MnBi_2_Te_4_ for axion insulator ($M=-0.2 \mathrm{eV}$) and normal insulator ($M=0.1 \mathrm{eV}$), respectively.


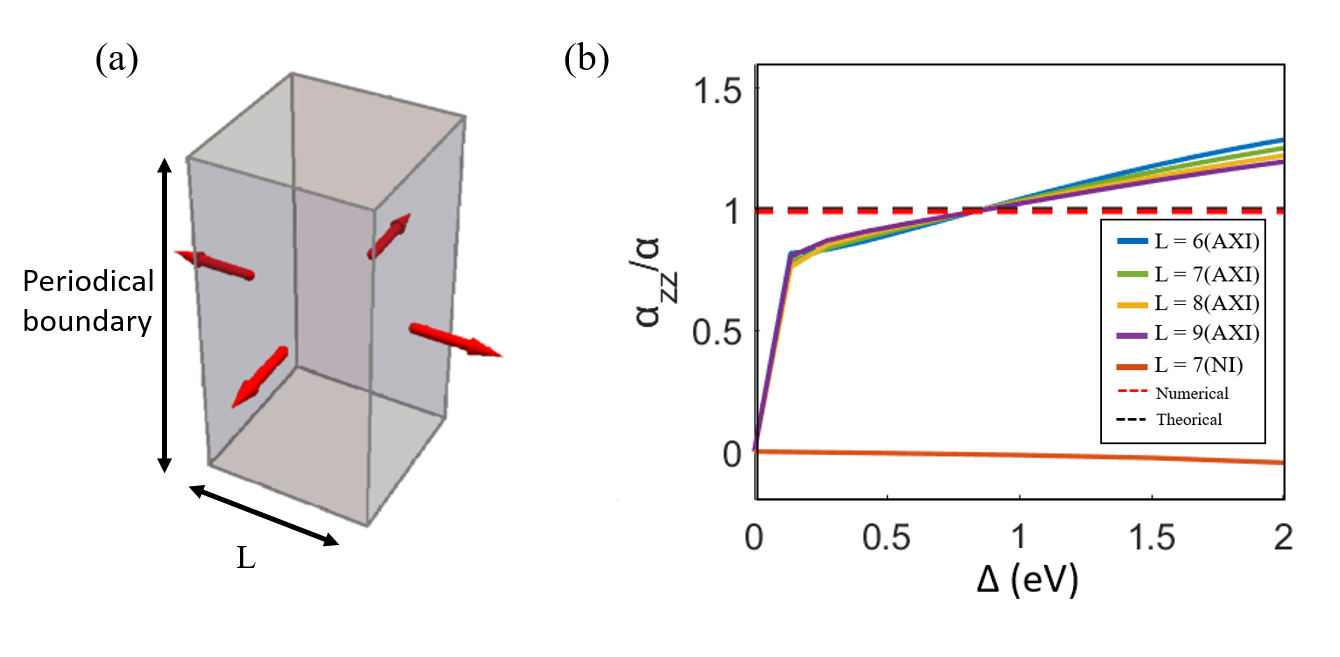


**Fig. S5.** (a) Sketch of cubic lattice 3D TI with surface magnetization normal to the side surfaces. (b) Results of the magnetoelectric coefficient as a function of the exchange strength $\Delta$ in this model in distinct phases.

We next consider the model Hamiltonian of 3D time-reversal invariant topological insulator (TI) written in a cubic lattice [3]

$$\mathcal{H}_{TI}=v\sum_{i=1}^{3} \sin k_{i}\alpha_{i}+\left[ mv^{2}-2B\sum_{i=1}^{3} \left( 1-\cos k_{i} \right) \right]\beta\left( S3 \right)$$

where $v=1, \alpha_{i}=s_{1}\otimes\sigma_{i}$, and $\beta=s_{3}\otimes\sigma_{0}$. The axion field $\theta=0$ or $\pi$ is guaranteed by both inversion $\mathcal{P=}s_{0}\otimes\sigma_{3}$ the time-reversal symmetry $\mathcal{T=}\left( -is_{2}\otimes\sigma_{0} \right)K$ ($K$ is the complex conjugation). With the help of the $\mathbb{Z}_{2}$ index of the strong TI phase, this system has a nontrivial bulk topology if $0<m<4B$ or $8B<m<12B$, accompanied with gapless helical modes on surfaces.

Considering a cubic 3D TI with periodical boundary condition in $z$ direction exposed to surface magnetization normal to the side surfaces [Fig. S5(a)], we expect that the surface states are gapped due to the broken of time-reversal symmetry on the surface, leading to a AXI phase once $\theta=\pi$ in bulk. Notice that the magnetic exchange coupling breaks the time-reversal symmetry, but the tetragonal geometry preserves the inversion symmetry that ensure the quantized $\theta=\pi$ in bulk. The numerical calculations of the magnetoelectric response of AXI and NI phases are displayed in Fig. S5(b). In AXI phase, the magnetoelectric response $\alpha_{zz}$ approaches to $\alpha_{0}=e^{2}/2hc$, while such a response vanishes in NI phase. These results support our speculation that $\alpha_{\mathrm{zz}}$ would quantize to $\alpha$ in a finite and large size axion insulator with all the surface gapped but preserved the symmetry (e.g., inversion symmetry) that protects the bulk axion field.

# **3. Transition between surface hybridization gap and magnetic gap**

For the FM MnBi_2_Te_4_ model of triangular prism geometry, the side surface gaps consist of two contributions, originated separately from the quantum confinement and magnetization. The quantum confinement hybridizes the surface states at different side surfaces if the side length $L$ is relatively short, bring no surface Hall conductivity. In contrast, the magnetic gap introduces mass term in surface Dirac mode, giving rise to half-quantized surface Hall effect and topological magnetoelectronic effect. By tuning the side length $L$ or the strength of the surface magnetic exchange coupling, the dominate origin of the surface gap will change from one to the other.

To characterize the transition of the surface gap, we adopt the local Chern marker to trace the topological property of the surface states. The Chern marker at each $\mathbf{r}$ normal to the (010) surface is defined as [4,5]

$$C_{zx}\left( \mathbf{r} \right)=-4\pi\mathrm{Im}\left\langle\mathbf{r} | \hat{P}\hat{z}\hat{Q}\hat{x}\hat{P} | \mathbf{r} \right\rangle\left( S4 \right)$$

where $\hat{x}$ and $\hat{z}$ are position operators, $\hat{P}$ is the projector on the occupied states, and $\hat{Q}=1-\hat{P}$. The numerical result of $C_{zx}$ at the side surface of the triangular prism is shown in Fig. S6. In no exchange limit $\Delta=0$ the surface gap is contributed completely by hybridization effect to give zero Chern marker. As the magnetic exchange is turned on, finite $C_{zx}$ and surface Hall effect gradually emerge. The slop of the Chern marker increases along with the enlargement of the side length, indicating the magnetic effect dominates the hybridization effect more easily in large system. For $L\gtrsim20$ and $\Delta\gtrsim40 \mathrm{meV}$, the surface Chern marker is stabilized to around 0.3 but not up to the half-quantized value 0.5. We attribute it to the inevitable hybridization near the hinges of the triangular prism. Notice that even in a cubic geometry, the local Chern markers at the surface states also deviate from 0.5 [6].


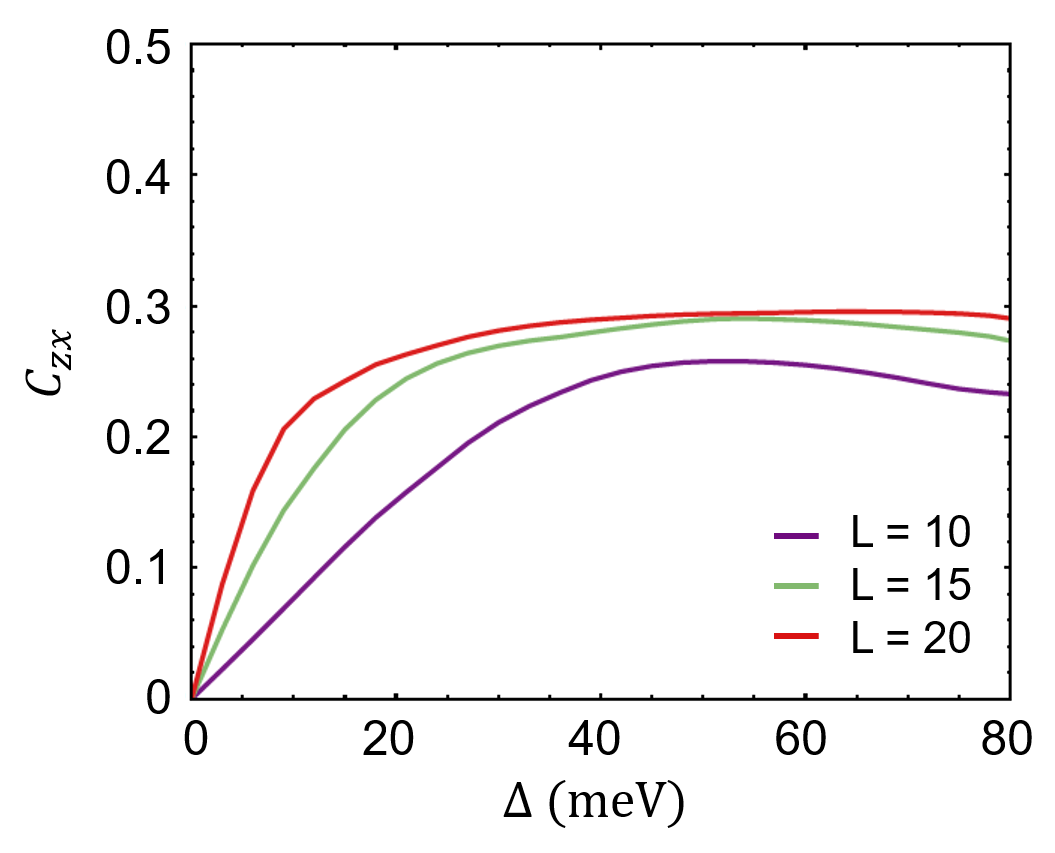


**Fig. S6.** Local Chern marker of the side surface as a function of side length $L$ and surface magnetic exchange $\Delta$.

# **4.** **Finite-layer model of** **ferromagnetic** **MnBi_2_Te_4_**

To verify the hinge mode in a finite-layer system terminated in $z$ direction, we in this section consider a FM MnBi_2_Te_4_ prism model with height $H$ and side length $L=8$, as displayed in Fig. S7(a). Model parameters are the same as those in Section 1. Energy levels of the finite-layer system with different height $H$ are shown as blue points in Fig. S7(b), in which the gray regions represent the bulk states determined by a periodic system with $k_{z}$. We examine that the energies of hinge states inside the energy gap are converged after $H\gtrsim10$. The spatial distribution of the hinge state is shown in Fig. 3 of the main text.


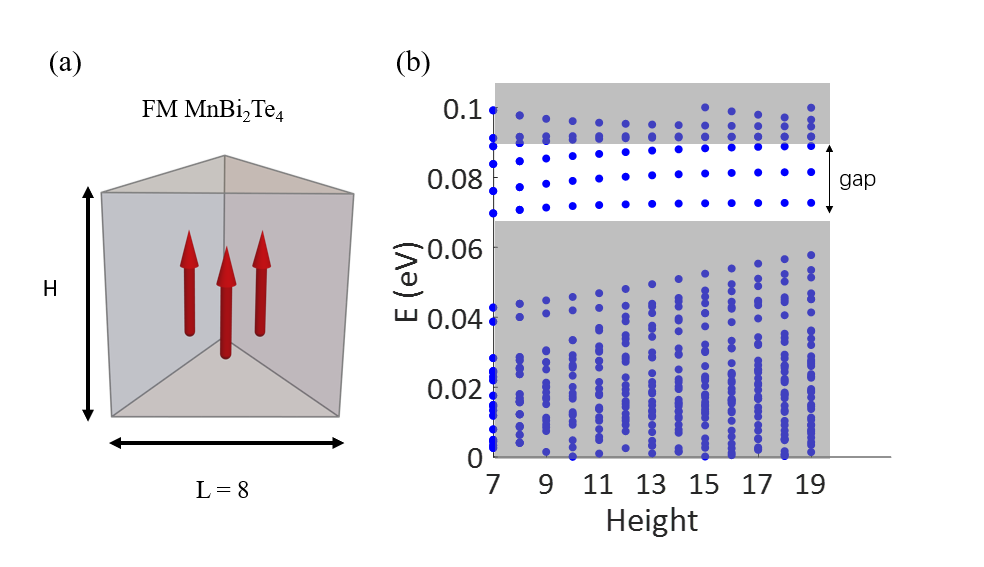


**Fig. S7.** (a) Sketch of finite-layer model of FM MnBi_2_Te_4_. (b) Energy levels of the finite-layer model with different height $H$, in which the energy gap is determined by the corresponding periodic system with $k_{z}$.

# **5. Magnetoelectric response in antiferromagnetic MnBi_2_Te_4_**

For numerical calculations, we use an effective stacked model for MnBi_2_Te_4_ [1]

$$\mathcal{H}_{AFM}=\left( \begin{matrix} h_{0}+\Delta\mathbf{m}\cdot\mathbf{s}\otimes\sigma_{0} & h_{AB} \\ h_{AB}^{\dagger} & h_{0}-\Delta\mathbf{m}\cdot\mathbf{s}\otimes\sigma_{0} \end{matrix} \right), \left( S5 \right)$$

with the intralayer term $h_{0}=\left[ \tilde{C}-\frac{4}{3}C_{2}\left( \cos k_{1}+\cos k_{2}+\cos k_{3} \right) \right]I_{4}+\frac{v}{3}\left( 2\sin k_{1}+\sin k_{2}+\sin k_{3} \right)\Gamma_{1}+\frac{v}{\sqrt{3}}\left( \sin k_{2}-\sin k_{3} \right)\Gamma_{2}+8w\left( -\sin k_{1}+\sin k_{2}+\sin k_{3} \right)\Gamma_{4}+\left[ \tilde{M}-\frac{4}{3}M_{2}\left( \cos k_{1}+\cos k_{2}+\cos k_{3} \right) \right]\Gamma_{5}$, and the interlayer term $h_{AB}=-2C_{1}\cos\left( \frac{k_{z}}{2} \right)I_{4}+2v_{z}\sin\left( \frac{k_{z}}{2} \right)\Gamma_{3}-2M_{1}\cos\left( \frac{k_{z}}{2} \right)\Gamma_{5}$. Definitions of parameters and basis are same as those in FM case in Section 1. The only difference is that a stagger magnetic orientation $\pm\mathbf{m}$ is replaced to produce the antiferromagnetic (AFM) order, in which we specifically set $\mathbf{m}=\left( 0,0,1 \right)$ for the A-type AFM order [see Fig. S8(a)]. Protected by the combined symmetry of time reversal $T$ and fractional translation $\tau_{1/2}$ along $z$ direction, the side surface states are gapless due to the bulk axion field $\theta=\pi$. However, once constructing an AFM MnBi_2_Te_4_ model in a triangular prism, side surface states are trivially gapped by the finite size effect. Following the same routine, we calculate the magnetoelectric response of the AFM system when the Fermi energy lies in gap. The numerical results in the AFM case are shown in Fig. S8(b) comparing with the results in the FM one. As expected, the AFM system does not produce finite TME effect, as the Hall current is absent for the finite-size-induced side surface gap.


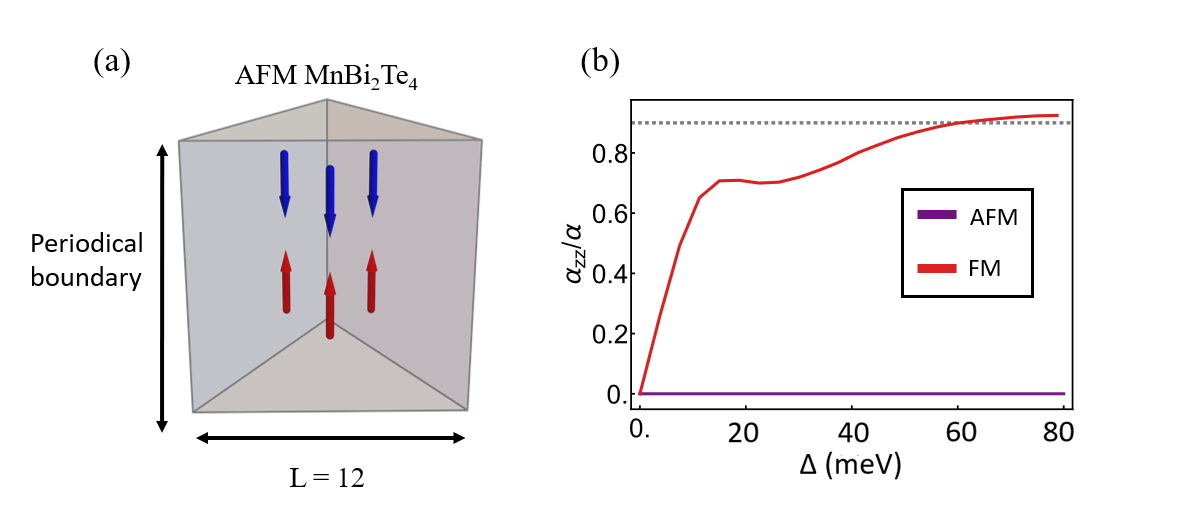


**Fig. S8.** (a) Sketch of triangular prism AFM MnBi_2_Te_4_. (b) Zero results of the magnetoelectric response of triangular prism AFM MnBi_2_Te_4_ under different exchange coupling $\Delta$, with the side length $L=12$ and translation symmetry along the *z*-direction (purple line), comparing with the finite result of the same system but with FM order.

**References**

[1] R.-X. Zhang, F. Wu, and S. Das Sarma, Möbius Insulator and Higher-Order Topology in MnBi_2n_Te_3n+1_, *Phys. Rev. Lett.* **124**, 136407 (2020).

[2] M. Gu, J. Li, H. Sun, Y. Zhao, C. Liu, J. Liu, H. Lu, and Q. Liu, Spectral signatures of the surface anomalous Hall effect in magnetic axion insulators, *Nat. Commun.* **12**, 3524 (2021).

[3] S.-Q. Shen, *Topological Insulators: Dirac Equation in Condensed Matter*, edited by S.-Q. Shen (Springer Singapore, Singapore, 2017),pp. 17.

[4] R. Bianco and R. Resta, Mapping topological order in coordinate space, *Phys. Rev. B* **84**, 241106 (2011).

[5] N. Varnava and D. Vanderbilt, Surfaces of axion insulators, *Phys. Rev. B* **98**, 245117 (2018).

[6] O. Pozo, C. Repellin, and A. G. Grushin, Quantization in Chiral Higher Order Topological Insulators: Circular Dichroism and Local Chern Marker, *Phys. Rev. Lett.* **123**, 247401 (2019).
